# NÍSIA TRINDADE LIMA: PRIDE OF FIOCRUZ, PRIDE OF BRASIL

**DOI:** 10.1590/S0104-59702023000100001en

**Published:** 2023-04-03

**Authors:** Marcos Cueto

**Affiliations:** i Scientific editor, researcher, Casa de Oswaldo Cruz/Fiocruz Rio de Janeiro – RJ – Brasil

In the past six years – after the coup against President Dilma Rousseff – most Brazilian researchers suffered episodes of aggression, intimidation and persecution from a government marked by the dismantlement of scientific research institutions and the criminal fragilization of sanitary, medical-environmental and social interventions based on science. By the end of 2022, an event brought us optimism: the designation of Nísia Trindade Lima as Minister of Health. As from January 2023, Lima is the first woman to head the ministry created in 1953, whose 50 predecessors were all men. She is a member of Luiz Inácio Lula da Silva’s democratic government, which has great possibility of revival and multiplication of the best moments of the recent history of Brazil.

The trust in Lima’s management rises not only from the fact that she is a woman. It confirms that social scientists and historians can contribute with their studies to public health policies and to the Brazilian Unified Health System (Sistema Único de Saúde, SUS). We know her intellectual capability because most of her researches and publications are in the field of the history of health. Her classical book *Um sertão chamado Brasil* studies the intellectual, sanitary and political assessments of a vast region of the country, analyses a metaphor of the fragmentation between more and less developed regions and reflects on the country’s social inequality (Lima, 2013).

Her remarkable trajectory provides Lima with the best credentials to face new challenges, thanks to her capability to articulate fruitful cross-sectoral dialogues between science, national politics and global health. Nísia Trindade Lima holds a master’s degree in political sciences and a doctoral degree in sociology from the Instituto Universitário de Pesquisas do Rio de Janeiro (currently Instituto de Estudos Sociais e Políticos). As from 1987, when entering Fiocruz, Lima developed special abilities by combining research and teaching with academic management. Her activities were developed at Casa de Oswaldo Cruz (COC), a Fiocruz unit specialized in studies on the history of sciences and health, scientific dissemination, and preservation of the memory and heritage of health. Between 1998 and 2005, Lima was a professor, head of the research department, vice-director and later COC director. In a recent issue of *História, Ciência, Saúde – Manguinhos*, we had the privilege of publishing her letter as invited editor, celebrating the 21th anniversary of the Postgraduate Program in History of Sciences and Health of COC. According to Lima, history is like “a window that opens to the past and to the present” and that “enables to think about the future as a product of historical imagination” (Lima, 2022, p.889).

Lima took her valuable experience to the Fiocruz Academic Press (Editora Fiocruz) and to Fiocruz Vice-presidency of Teaching, Information and Communication, where she was outstanding in the effort to insert Fiocruz in global health. Lima was elected president of the Foundation in 2017 – as the first woman to occupy the post – with ample support of sanitarians from Fiocruz and other Brazilian health institutions. In 2021, she was re-elected for a second term. With wisdom and sobriety, Lima led the actions to face the covid-19 pandemic. She made feasible the rapid construction of the Hospital Centre for Covid-19 Pandemic (Centro Hospitalar para a Pandemia de Covid-19) on Manguinhos campus, the organization of emergency actions among vulnerable populations, and the partnership of Fiocruz with Oxford University and the pharmaceutical company AstraZeneca for the production, in Brazil, of the vaccine against covid-19. Since then, the unity Bio-Manguinhos turned into one of the main Latin American vaccine production centers.

In her trajectory in Fiocruz, besides developing intellectual ability, political intelligence and professional experience, Lima conquered the admiration and respect from her colleagues and the scientific community. Among the numerous recognitions received, we highlight her election in December 2020 as a Full Member of the Brazilian Academy of Sciences in the category Social Sciences. Two years later, Lima was appointed as a Fellow of The World Academy of Sciences, an academic organization dedicated to the advancement of science in developing countries.

We trust that Lima is an extraordinary administrator and persuasive communicator to different audiences, capable of managing overwhelming challenges, such as facing the dismantlement of the SUS funding, the disruption of primary healthcare and the restoration of programs as the National Immunizations Program and the Popular Pharmacy Program of Brazil. Lima will certainly need to use all her qualities in the slippery, unstable terrain of contemporary politics. We are confident that her talent, competence and dedication will shortly confirm the title of this letter. Besides being a source of pride to all health historians, Oswaldo Cruz House and Fiocruz, we are certain that soon the Brazilian population will also take pride in Nísia Trindade Lima.

We wish to conclude this letter with a different and important subject related to the good practices of open science used by many journals of SciELO collection. From 2023 onwards, *História*, *Ciências, Saúde* – *Manguinhos* will adopt the continuous publication system (also known as continuous stream of publication). This system consists on the electronic publication of the journal’s articles, isolated or in small groups, as soon as they are approved and internally processed (linguistic revision, normalization, layout etc.), instead of publishing all articles of an issue at once. The continuous publication system enables greater agility in the dissemination of our authors’ researches and it will increase the journal’s visibility.

Hopefully, this editorial modernization and the good news about Nísia Trindade Lima’s taking office as Minister of Health represents the beginning of a promising year for the scientific community and for public health in Brazil.
